# Aspects of Juvenile and Adolescent Environment Predict Aggression and Fear in 12-Month-Old Guide Dogs

**DOI:** 10.3389/fvets.2016.00049

**Published:** 2016-06-22

**Authors:** James A. Serpell, Deborah L. Duffy

**Affiliations:** ^1^Department of Clinical Studies, School of Veterinary Medicine, University of Pennsylvania, Philadelphia, PA, USA

**Keywords:** dog, behavior, development, puberty, C-BARQ

## Abstract

Maturational changes in behavior, and the possible influence of the puppy-raising environment on behavioral development, were investigated in a total sample of 978 prospective guide dogs belonging to four different breeds/crosses. All dogs belonged to the same guide dog organization, and had been exposed to similar early environmental influences prior to being assigned to puppy-raising households at 7–8 weeks of age. Behavioral data were collected from puppy raisers when the dogs were 6 and 12 months old using the C-BARQ^©^, a standardized, validated, and widely used survey instrument that measures the frequency and/or severity of most common behavior problems in dogs. Information about the puppy-raising environment was obtained from puppy raisers using a standardized questionnaire shortly before the dogs were returned to the guide dog organization for training. Data were analyzed using both univariate and multivariate statistics (binary logistic generalized estimating equations modeling and generalized linear modeling). The findings demonstrated specific maturational changes in behavior between 6 and 12 months of age. In particular, German Shepherd dogs displayed an increase in stranger-directed aggression compared with the other breeds/crosses between 6 and 12 months of age. Several aspects of the puppy-raising environment were associated with puppies’ C-BARQ scores at 12 months of age. In particular, growing up in households with more experienced puppy raisers, and in the company of at least one other dog, were both associated with positive effects on a number of puppy behaviors. By contrast, puppies that had been frightened by a person or threatened by another unfamiliar dog showed significantly worse scores for fear of strangers and dogs, respectively. Being frightened by a person, being reared by less experienced puppy raisers, and/or in households without other pets were associated with less successful training outcomes. The relevance of these findings to current guide dog breeding and husbandry practices is discussed.

## Introduction

According to Scott and Fuller (1), the juvenile period in dogs runs from ~12 weeks (the postulated end of the socialization period) until 6 months of age or later, corresponding to the onset of sexual maturation (i.e., puberty). Extensive studies in rodents and both human and non-human primates have demonstrated that changes in circulating gonadal hormones during the adolescent period have dramatic effects on brain development and behavior in mammals (2, 3). Despite the likelihood of similar developmental influences in adolescent dogs (between ~6 months and 1–2 years of age depending on the individual and breed), this period is probably the most poorly studied in terms of its effects on adult behavior (4). Anecdotal evidence certainly suggests that experiences during the juvenile and adolescent periods appear exert long-term effects on behavior in dogs (5–8), and one recent study of military working dogs found a positive association between dogs’ overall scores for working ability and the amount of time they were left at home alone during this period (9). In rodents, exposure to enriched environments around puberty has been found to erase the negative effects of early life stress on the development of the HPA axis (10), but whether similar effects occur in dogs is currently unknown.

Purpose-bred guide dogs provide a useful model for studying canine behavioral development during this period for several reasons. First, they usually comprise a relatively small number of different breed types – typically Labrador retriever, golden retriever, German Shepherd dog (GSD), and Labrador × golden retriever crosses – and this enables both within-breed and across-breed comparisons. Second, these dogs remain sexually intact until around 14–18 months of age so the potentially confounding effects of the loss of gonadal hormones on brain development and behavior due to surgical sterilization are effectively eliminated. Third, up to the age of 7–9 weeks, the puppies produced within any given organization will experience similar and relatively consistent early rearing environments, thus helping to reduce the effects of differential environmental influences occurring during early development. Finally, from roughly 2 to 14 months of age, guide dog puppies are reared in the households of volunteer puppy raisers (i.e., individuals and families who foster guide dog puppies) where they are exposed to variable environments, thereby providing an opportunity to explore the possible effects of this variability on their behavioral development during the juvenile and adolescent periods. Here, we present findings based on behavioral data collected from puppy raisers associated with a single guide dog organization when the dogs were 6 and 12 months of age, respectively. In addition to exploring maturational changes in behavior in dogs between 6 and 12 months (i.e., assumed to represent juvenile and adolescent dogs, respectively), we also investigate associations between behavioral outcomes at 12 months of age, and during subsequent training, and antecedent environmental factors and events occurring during the juvenile and adolescent periods.

## Materials and Methods

### Sample

For the study of maturational changes in behavior, a sample 741 young guide dogs (376 males and 365 females) were used, which included a mix of four breed types (*N* = 226 GSDs, 145 golden retrievers, 210 Labrador retrievers, and 160 golden × Labrador crosses). For the analysis of environmental influences on behavioral development, the sample of dogs comprised 472 males and 506 females (*N* = 978 total) and included the same mix of breed types (*N* = 276 GSDs, 207 golden retrievers, 296 Labrador retrievers, and 219 golden × Labrador crosses). All of the dogs belonged to a single, US-based guide dog organization.

### Data Collection

Behavioral data were provided by the participating guide dog organization that routinely collects behavioral information on each dog from puppy raisers when the dogs are ~6 and 12 months old using the C-BARQ^©^, a standardized, validated, and widely used survey instrument. The C-BARQ^©^ allows dog owners and handlers to describe the frequency and/or severity of most common behavior problems in dogs (11, 12). The specific behavioral variables that were examined included four different types of aggression and five different types of fear/anxiety (Table 1).

**Table 1:** **C-BARQ aggression and fear/anxiety variable descriptions (numbers of questionnaire items in parentheses)**.

C-BARQ trait	C-BARQ variable description (number of questionnaire items)
Stranger-directed aggression	Severity of threatening or aggressive responses to strangers approaching or invading the dog’s or owner’s personal space, territory, or home range (10)
Dog-directed aggression	Severity of threatening or aggressive responses when approached directly by unfamiliar dogs (4)
Owner-directed aggression	Severity of threatening or aggressive responses to the owner or other members of the household when challenged, manhandled, stared at, stepped over, or when approached while in possession of food or objects (8)
Dog rivalry	Severity of aggressive or threatening responses to other familiar dogs in the same household (4)
Stranger-directed fear	Severity of fearful or wary responses when approached directly by strange or unfamiliar people (4)
Dog-directed fear	Severity of fearful or wary responses when approached directly by unfamiliar dogs (4)
Non-social fear	Severity of fearful or wary responses to sudden or loud noises, traffic, and unfamiliar objects, and situations (6)
Separation-related behavior	Frequency of vocalizing and/or destructive behavior when separated from the owner, including autonomic signs of anxiety – restlessness, loss of appetite, trembling, and excessive salivation (8)
Touch sensitivity	Severity of fearful or wary responses to potentially painful or uncomfortable procedures, including bathing, grooming, nail-clipping, and veterinary examinations (4)

Information on the dogs’ household characteristics and environmental exposures, was obtained from the “Puppy-Raiser Report” questionnaire (see Supplementary Materials), which all puppy raisers are asked to complete routinely prior to returning their dogs to the parent organization for training at ~14 months of age. This survey provides relatively detailed information about the characteristics of the household, the experience level of the puppy raisers, and the puppy’s level of exposure to a wide variety of potential environmental stressors and stimuli.

In addition, information on each dogs’ eventual disposition – i.e., whether it became a successful working dog, a breeding dog, a dog that was released for health reasons, or one that was released for behavioral reasons – was also provided by the participating guide dog organization.

As the owner of both the dogs and the data, the participating guide dog organization provided written consent for the use of this information in the current study.

### Statistical Analyses

For the determination of maturational changes in aggressive and fearful behavior, both 6- and 12-month C-BARQ scores were used, and the effects were analyzed with binary logistic generalized estimating equations modeling, with dichotomized C-BARQ scores as dependent variables. Because scores for all aggression and fear/anxiety variables were strongly skewed, C-BARQ scores were dichotomized such that a score of 0 indicated the absence of the behavior and all scores above 0 were recoded as indicating the presence of the behavior. Predictor variables included age at evaluation as a within-subjects variable, and breed and sex as between-subjects variables. Interactions between age at evaluation with either breed or sex were examined and retained in the final models, if significant.

To investigate the effects of environmental factors on behavior, preliminary analyses were conducted using non-parametric tests (Mann–Whitney U, Kruskal–Wallis, and Chi-square tests) to identify the main household and experiential factors associated with behavioral differences at 12 months of age and later success in training (“outcomes”) (see Supplementary Tables). Generalized linear models (with logit link function) were then used to identify the key environmental variables associated with training outcomes and the presence of aggression and fear/anxiety while accounting for the effects of breed differences in behavior. A backwards elimination procedure was utilized, removing predictor variables with *p*-values below 0.05. Cases with missing values (*N* ≤ 50) for any predictors were excluded from the analysis on a model by model basis. As before, aggression and fear/anxiety C-BARQ subscales were dichotomized, with 0 indicative of the absence of the behavior and all scores greater than 0 indicative of the presence of the behavior.

## Results

### Maturational Changes

**Figure 1:**
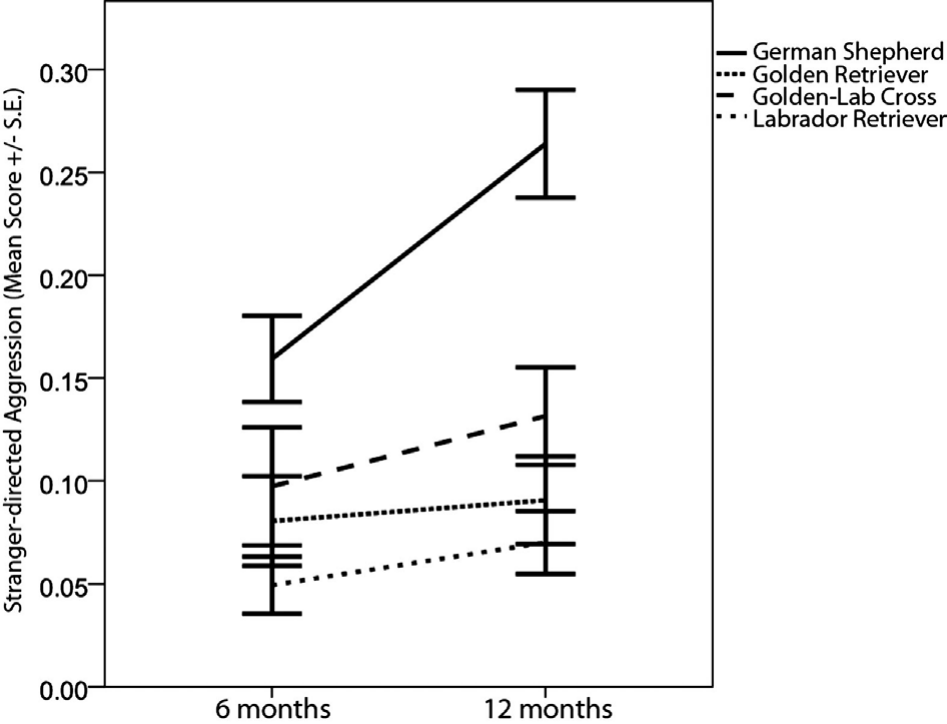
**Maturational changes in average scores (±SE) for the C-BARQ factor “stranger-directed aggression” between 6 and 12 months in the four guide dog breeds/breed types**.

Comparisons of young guide dogs’ C-BARQ scores at 6 and 12 months of age revealed interesting developmental changes, which sometimes varied by breed or sex. GSDs, a breed that has been selected historically for guarding or “protective” behavior, showed increases in “stranger-directed aggression” between 6 and 12, while the other three breeds showed only minimal increases or none at all (Table 2; Figure 1). During the same time frame, decreases were observed in “owner-directed aggression,” “separation-related problems,” and “non-social fear,” though the latter was only detectable among females (Table 2).

**Table 2:** **Results of binary logistic generalized estimating equations modeling to examine maturational changes in C-BARQ traits**.

C-BARQ traits and predictors^a^	Wald Chi-square	df	*p*-value	OR	95% CI
**Stranger-directed aggression**					
*Age at evaluation*	14.13	1	0.0002^b^	–	–
Age = 12 months	2.67	1	0.102	1.45	0.93, 2.45
*Sex = Female*	0.21	1	0.648	0.93	0.68, 1.27
*Breed*	67.96	3	<0.0001^b^	–	–
German Shepherd	22.62	1	<0.0001^b^	3.70	2.16, 6.35
Golden retriever	2.83	1	0.092	1.71	0.92, 3.19
LabGolden Cross	2.13	1	0.144	1.59	0.85, 2.97
*Breed × Age*	10.01	3	0.018	–	–
German Shepherd at 12 months	3.21	1	0.073	1.68	0.95, 2.97
Golden retriever at 12 months	1.49	1	0.223	0.67	0.36, 1.27
Lab × Golden Cross at 12 months	0.42	1	0.838	1.07	0.56, 2.04
**Owner-directed aggression**					
*Age = 12 months*	14.31	1	0.0002^b^	0.69	0.58, 0.84
*Sex = female*	0.04	1	0.839	0.97	0.58, 1.30
*Breed*	2.43	3	0.488	–	–
German Shepherd	0.98	1	0.321	1.22	0.82, 1.80
Golden retriever	0.84	1	0.360	1.22	0.79, 1.89
Lab × Golden Cross	0.08	1	0.782	0.94	0.61, 1.45
**Dog-directed aggression/fear**					
*Age = 12 months*	0.48	1	0.489	0.92	0.71, 1.17
*Sex = female*	0.03	1	0.874	0.98	0.71, 1.33
*Breed*	9.26	3	0.026	–	–
German Shepherd	4.15	1	0.042	1.54	1.02, 2.33
Golden retriever	1.38	1	0.241	1.30	0.84, 2.03
Lab × Golden Cross	8.53	1	0.003^b^	1.96	1.25, 3.09
**Dog rivalry**					
*Age at evaluation*	0.06	1	0.804	0.97	0.78, 1.21
*Sex = Female*	0.08	1	0.783	1.04	0.78, 1.40
*Breed*	15.11	3	0.002^b^	–	–
German Shepherd	13.55	1	<0.0001^b^	2.10	1.42, 3.12
Golden retriever	1.08	1	0.299	1.28	0.81, 2.03
Lab × Golden Cross	4.59	1	0.032	1.64	1.04, 2.58
**Stranger-directed fear**					
*Age = 12 months*	0.001	1	0.981	1.00	0.74, 1.34
*Sex = Female*	0.002	1	0.962	0.96	0.65, 1.50
*Breed*	6.57	3	0.087	–	–
German Shepherd	2.02	1	0.156	1.48	0.86, 2.52
Golden retriever	0.55	1	0.458	0.77	0.38, 1.54
Lab × Golden Cross	2.71	1	0.100	1.61	0.91, 2.85
**Touch sensitivity**					
*Age = 12 months*	0.64	1	0.425	1.29	0.99, 1.67
*Sex = Female*	0.39	1	0.531	1.09	0.78, 1.53
*Breed*	18.71	3	<0.0001^b^	–	–
German Shepherd	15.87	1	<0.0001^b^	2.15	1.48, 3.13
Golden retriever	0.62	1	0.432	1.17	0.79, 1.75
Lab × Golden Cross	6.77	1	0.009	1.69	1.14, 2.50
**Separation-related problems**					
*Age = 12 months*	7.43	1	0.006^b^	0.74	0.59, 0.92
*Sex = Female*	0.67	1	0.412	0.88	0.66, 1.19
*Breed*	9.91	3	0.019	–	–
German Shepherd	7.60	1	0.006^b^	1.73	1.17, 2.55
Golden retriever	0.04	1	0.842	0.96	0.63, 1.45
Lab × Golden Cross	1.09	1	0.297	1.24	0.83, 1.85
**Non-social fear**					
*Age at evaluation*	2.52	1	0.112	–	–
Age = 12 months	0.30	1	0.585	1.11	0.77, 1.60
*Sex*	0.12	1	0.728	–	–
Sex = Female	2.54	1	0.111	1.45	0.92, 2.30
*Breed*	10.74	3	0.013	–	–
German Shepherd	2.22	1	0.136	1.35	0.91, 2.00
Golden retriever	10.50	1	0.001^b^	2.37	1.41, 3.98
Lab × Golden Cross	2.35	1	0.125	1.41	0.91, 2.19
*Sex (Females) × Age (12 months)*	5.49	1	0.019	0.53	0.31, 0.90

*^a^Results in italics are model effects, non-italicized results are parameter estimates. Reference groups were as follows: age = 6 months, sex = male, and breed = Labrador retriever*.

*^b^Statistically significant with Bonferroni correction for multiple comparisons (0.05/8 tests = 0.006)*.

### Environmental Influences on Behavior

**Figure 2:**
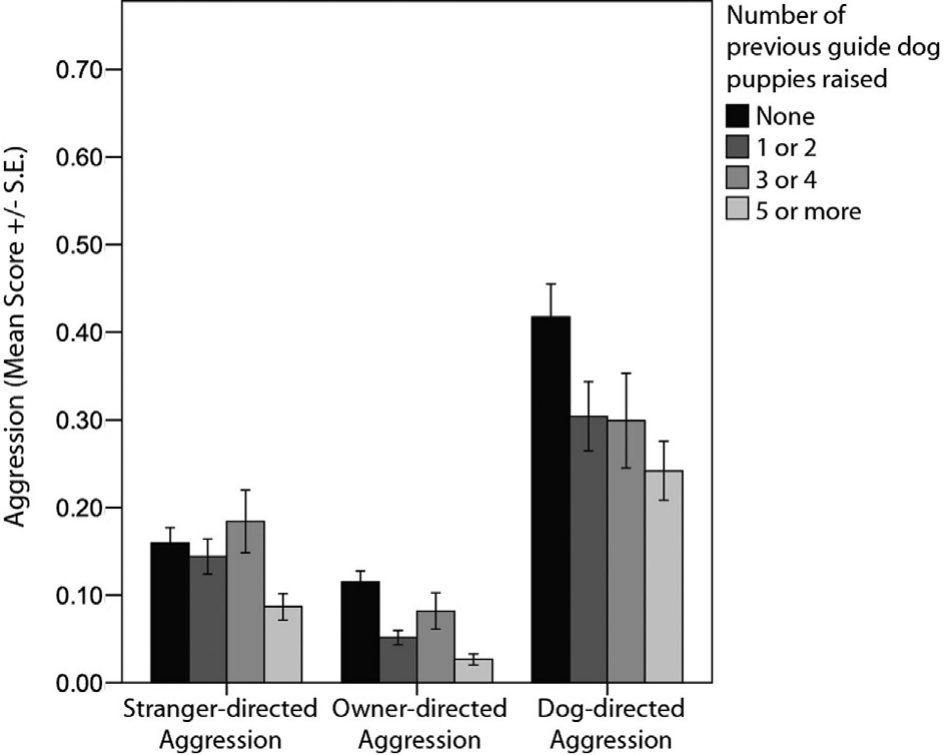
**Comparison of average C-BARQ scores (±SE) for “stranger-directed aggression,” “owner-directed aggression,” and “dog-directed aggression” in relation to the number of previous guide dog puppies raised by the puppy raiser**.

**Figure 3:**
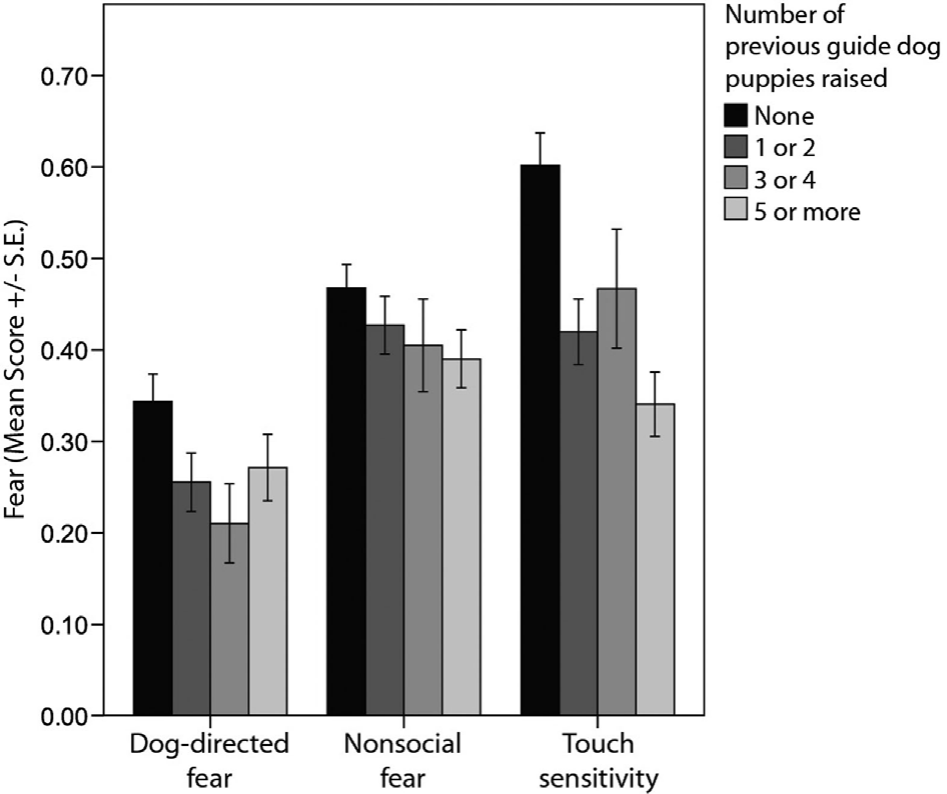
**Comparison of average C-BARQ scores (±SE) for “dog-directed fear,” “non-social fear,” and “touch sensitivity” in relation to the number of previous guide dog puppies raised by the puppy raiser**.

Puppy-raisers’ prior experience with raising guide dogs was associated with a number of behavioral differences in guide dogs at 12 months of age, including “owner-directed aggression,” “stranger-directed aggression,” “dog-directed aggression,” “dog-directed fear,” “non-social fear,” and “touch sensitivity” (Tables 3 and 4; Figures 2 and 3). Likewise, there was also a tendency for an association between the number of previous guide dog puppies raised by the puppy raiser and the likelihood that a puppy would successfully complete training (Table 5).

**Table 3:** **Results of generalized linear modeling with logit link function to examine environmental factors associated with C-BARQ aggression factors**.

C-BARQ trait and predictors	*p*-value	Odds ratio	Confidence interval	Est. marginal means
**Owner-directed aggression^a^**				
Number of puppies raised	<0.0001^b^			
None	–	–	–	0.41
1 or 2	0.0002^b^	0.47	0.32, 0.70	0.25
3 or 4	0.078	0.61	0.35, 1.05	0.30
5 or more	<0.0001^b^	0.29	0.16, 0.44	0.16
Other pets	<0.0001^b^			
None	–	–	–	0.34
Other dogs	<0.0001^b^	0.35	0.23, 0.54	0.015
Other pets but no dogs	0.80	1.06	0.67, 1.69	0.35
**Stranger-directed aggression**				
Breed	<0.0001^b^			
German Shepherd	–	–	–	0.62
Golden retriever	<0.0001^b^	0.23	0.15, 0.35	0.27
Lab × Golden Cross	<0.0001^b^	0.32	0.21, 0.48	0.34
Labrador retriever	<0.0001^b^	0.20	0.13, 0.30	0.25
Number of puppies raised	0.007			
None	–	–	–	0.41
1 or 2	0.65	0.92	0.63, 1.33	0.39
3 or 4	0.97	0.99	0.60, 1.64	0.40
5 or more	0.001^b^	0.51	0.34, 0.77	0.26
Puppy threatened by dog	0.004^b^			
No	–	–	–	0.31
Yes	0.004^b^	1.61	1.16, 2.34	0.42
**Dog-directed aggression**				
Breed	<0.0001^b^			
German Shepherd	–	–	–	0.60
Golden retriever	<0.0001^b^	0.23	0.14, 0.36	0.26
Lab × Golden Cross	0.004^b^	0.46	0.30, 0.071	0.41
Labrador retriever	<0.0001^b^	0.25	0.16, 0.37	0.27
Number of puppies raised	0.015			
None	–	–	–	0.46
1 or 2	0.008	0.60	0.41, 0.87	0.34
3 or 4	0.23	0.72	0.43, 1.22	0.39
5 or more	0.007	0.55	0.36, 0.85	0.32
Other dogs in the household	0.02			
No	–	–	–	0.42
Yes	0.02	0.68	0.49, 0.95	0.33
Teenagers in the household	0.03			
No	–	–	–	0.34
Yes	0.03	1.44	1.04, 2.00	0.42

*^a^Breed effects were controlled for by inclusion in the model but did not reach significance*.

*^b^Statistically significant with Bonferroni correction (eight GLM tests with family-wise error rate of 0.05)*.

**Table 4:** **Results of generalized linear modeling with logit link function to examine environmental factors associated with C-BARQ fear factors**.

C-BARQ trait and predictors	*p*-value	Odds ratio	Confidence interval	Est. marginal means
**Stranger-directed fear^a^**				
Puppy frightened by a person	<0.0001^b^			
No	–	–	–	0.08
Yes	<0.0001^b^	4.60	2.45, 8.39	0.29
**Dog-directed fear^a^**				
Number of puppies raised	0.05			
None	–	–	–	0.54
1 or 2	0.04	0.69	0.48, 0.99	0.45
3 or 4	0.02	0.54	0.33, 0.89	0.39
5 or more	0.07	0.68	0.47, 1.03	0.45
Puppy threatened by dog	0.003^b^			
No	–	–	–	0.40
Yes	0.003^b^	1.59	1.17, 2.18	0.51
Other dogs in household	0.06			
No	–	–	–	0.49
Yes	0.06	0.75	0.55, 1.01	0.43
**Non-social fear**				
Breed	0.033			
German Shepherd	–	–	–	0.78
Golden retriever	0.43	1.22	0.75, 1.97	0.81
Lab × Golden Cross	0.21	0.74	0.47, 1.18	0.72
Labrador retriever	0.04	0.65	0.43, 0.99	0.70
Number of puppies raised	0.037			
None	–	–	–	0.82
1 or 2	0.03	0.64	0.43, 0.97	0.74
3 or 4	0.06	0.60	0.35, 1.02	0.73
5 or more	0.009	0.57	0.38, 0.87	0.72
**Touch sensitivity**				
Breed	0.002^b^			
German Shepherd	–	–	–	0.69
Golden retriever	0.001^b^	0.48	0.31, 0.73	0.51
Lab × Golden Cross	0.35	0.81	0.52, 1.27	0.64
Labrador retriever	0.003^b^	0.55	0.37, 0.82	0.55
Number of puppies raised	<0.0001^b^			
None	–	–	–	0.73
1 or 2	0.001^b^	0.53	0.37, 0.78	0.58
3 or 4	0.005^b^	0.48	0.29, 0.80	0.56
5 or more	<0.0001^b^	0.43	0.29, 0.64	0.52
Teenagers in household	0.038			
No	–	–	–	0.56
Yes	0.04	1.39	1.02, 1.91	0.64

*^a^Breed effects were controlled for by inclusion in the model but did not reach significance*.

*^b^Statistically significant with Bonferroni correction (eight GLM tests with family-wise error rate of 0.05)*.

**Table 5:** **Results of generalized linear modeling of environmental variables (predictors) and training outcomes**.^a^

Environmental variables	*p*-value	Odds ratio	Confidence interval	Est. marginal means
Puppy ever frightened by a familiar or unfamiliar person	0.008			
No	–	–	–	0.57
Yes	0.008	0.446	0.245, 0.812	0.37
Other pets in the household	0.040			
None	–	–	–	0.38
Other dogs	0.047	1.525	1.005, 2.315	0.49
Other pets but no dogs	0.013	1.884	1.144, 3.103	0.54
Number of guide dog puppies raised^b^	0.054	1.032	0.999, 1.065	–

*^a^Training outcomes reference = released from training*.

*^b^Continuous variable*.

**Figure 4:**
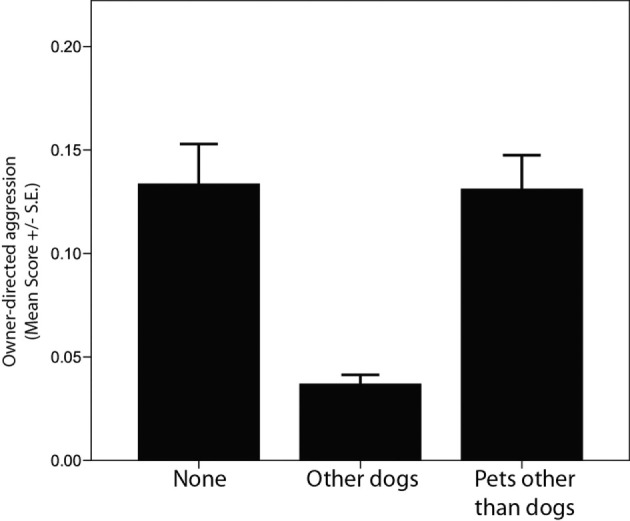
**Comparison of average C-BARQ scores (±SE) for “owner-directed aggression” among dogs raised in households with no other pets, with other dogs, and with pets other than dogs**.

Another contributor to behavioral differences observed among dogs at 12 months of age was whether a puppy was raised in a home with other dogs. Growing up in homes with other dogs was associated with significantly lower levels of aggression directed toward household members (Table 3; Figure 4), an effect specific to other dogs in the home and not the presence of pets other than dogs. Other dogs in the household was also associated with lower levels of aggression and fear directed toward unfamiliar dogs (Tables 3 and 4), but these effects were not significant after corrections for multiple comparisons were made. Other pets in the household, whether dogs or pets other than dogs, were associated with improved likelihood of succeeding in training (Table 5).

**Figure 5:**
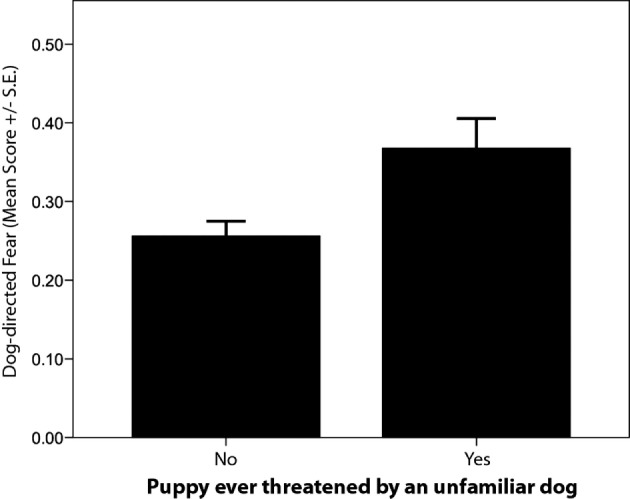
**Comparison of average C-BARQ scores (±SE) for “dog-directed fear” in guide dogs reported to have been threatened by an unfamiliar dog**.

**Figure 6:**
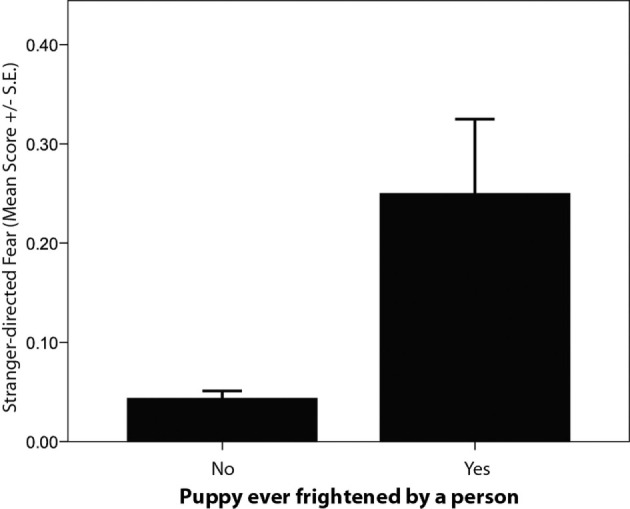
**Comparison of average C-BARQ scores (±SE) for “stranger-directed fear” in guide dogs reported to have been frightened by a person**.

Particular frightening or traumatic events during the puppy-raising period were associated with differences in C-BARQ scores for some behaviors. Specifically, puppies that were reported as having been attacked or threatened by another (unfamiliar) dog displayed significantly higher “dog-directed fear” and “stranger-directed aggression” at 12 months of age compared to those that had not had that experience (Tables 4 and 5; Figure 5). Likewise, puppies that were reported as having been frightened by a familiar or unfamiliar person showed significantly higher levels of “stranger-directed fear” (Table 4; Figure 6). Having been frightened by a person was also associated with lower likelihood of successfully completing training (Table 5).

The presence of teenagers in the household was associated with greater tendencies for dogs to display “dog-directed aggression” and “touch sensitivity” (Tables 4 and 5), though these effects were no longer significant with correction for multiple comparisons.

## Discussion

This analysis of guide dog development has been able to partially isolate the effects of the post-socialization rearing environment from those arising from events and exposures occurring during the earlier prenatal, neonatal, and primary socialization periods. Although the conclusions that can be drawn from retrospective reports are inevitably somewhat limited, the findings support the view that certain events and experiences occurring during the juvenile and adolescent periods are predictive of behavioral outcomes at 12 months of age, and that different breeds appear to respond differently to these experiences. Whether such associations are restricted to particular sensitive periods, or are equally likely to occur at any time throughout this stage of development, is unknown. However, the current analysis of maturational changes suggests that some dogs are predisposed to develop aggressive reactions to unfamiliar individuals between roughly 6 and 12 months of age, and that this predisposition is stronger in the German Shepherd breed than in the other three breed types investigated. This finding is of interest in light of anecdotal reports of a phase of heightened sensitivity to territorial stimuli in wolves at around 4–5 months age [see Ref. (6–8)], and also indicates that this onset of defensive behavior is not necessarily associated with a corresponding increase in fear of strangers. This implies that the aggressive element of defensive/territorial behavior develops separately from the fearful component, and suggests that such fears may become established earlier in life. Since owner-directed aggression decreases in all breeds over the same period, the findings further suggest that the rise in stranger-directed aggression in German Shepherds is not due to an overall increase in aggressiveness, but rather a specific response to unfamiliar persons. These results also reinforce the view that it is the *interaction* between a dog’s genetic background and its developmental environment that ultimately determines the adult behavioral phenotype.

With respect to developmental effects of specific events and exposures during this period, the findings illustrate the important role that the puppy-raiser’s puppy-rearing experience plays in determining behavioral outcomes, as well as the value of social exposure to other dogs in the household. Both of these variables are potentially modifiable either through education, in the case of experience, or through the deliberate recruitment of puppy raisers from among existing experienced dog owners, and such efforts or interventions would be likely to have a significant positive impact on the performance of future working dogs, especially for those breeds that are predisposed to developing problems.

The results also draw attention to the apparent sensitivity of dogs at this age to the effects of traumatic events or experiences, such as being frightened by a person, or being threatened or attacked by another dog. Such findings reinforce the view that puppies and young dogs are sensitive to aversive social encounters long after the ostensible end of the socialization period (i.e., 12 weeks), and that such encounters may have long-term negative consequences for behavior. Unfortunately, because of the correlational nature of these outcomes, it is not possible to determine whether these dogs became more fearful and/or aggressive as a direct consequence of their experiences or if they were more likely to be traumatized by an aversive encounter due to a pre-existing disposition toward fearfulness.

While some of the current results are unsurprising – for example, the reduced scores for the C-BARQ variables dog-directed fear and dog-directed aggression among puppies reared with other dogs – others are less easy to interpret. The reduction in “owner-directed aggression” in multi-dog households could perhaps be attributed to puppies developing enhanced social skills as a consequence of entering households at the bottom of established hierarchies. A study of pet dogs in Taiwan also found lower rates of owner-directed aggression among dogs living in multi-dog households (13), and an early study of guide dog puppies found that those reared in homes with other dogs were less distracted by other unfamiliar dogs when tested on walks at 6 and 12 months of age (14). By contrast, an Australian study of GSDs found that the presence of another dog in the household had a deleterious effect on dogs’ scores on tests of fearfulness, social attraction and “dominance” (15), while a recent study of guide dog puppies in the UK found a positive association between puppies’ ratings for energy level and distractibility and the number of dogs in the puppy-raising household (16).

With respect to puppy-raiser experience, these results are in agreement with those of previous studies that found that pet dogs belonging to first-time dog owners were more likely to display owner-directed aggression than those of more experienced owners (17, 18). One interpretation of such findings is that dog owners learn from experience how to prevent the development of some canine behavior problems, and that they get better at doing this with each successive dog owned. Similar effects may also account for the reduced energy and distractibility ratings of puppies reared by more experienced “puppy walkers” in a recent UK study of guide dogs (16). It is also possible that puppy-raising experience enhances owners’/handlers’ ability to “read” and react to canine emotions. Previous research has shown, for example, that increasing levels of experience with dogs is associated with improved human ability to recognize canine emotions, such as fear (19).

Most previous studies of canine behavioral development have tended to focus exclusively on the traditional 3- to 12-week socialization period, and this has perhaps discouraged investigators from exploring the possible consequences of later exposure to biologically relevant events and stimuli. Further investigations that emphasize the precise timing and quality of experiences within this developmental period would be likely to yield valuable information regarding appropriate husbandry practices for dogs of this age.

## Ethics

The study was approved by the Privately-owned Animals Subcommittee of the University of Pennsylvania Institutional Animal Care and Use Committee. The guide dogs and the survey data that were used in this study were owned by The Seeing Eye, Inc., Morristown, NJ, USA. This organization provided written consent for the use of all survey data pertaining to these dogs.

## Author Contributions

JS initiated the research, proposed the idea, and drafted the paper. DD performed the analysis, wrote up the results, and edited the paper.

## Conflict of Interest Statement

The research was conducted in the absence of any commercial or financial relationships that could be construed as a potential conflict of interest. The copyright for the C-BARQ instrument that was used as the source of behavioral information in the current study is owned jointly by the corresponding author (JS) and the University of Pennsylvania.
