# Supplementary material for: Aspects of Juvenile and Adolescent Environment Predict Aggression and Fear in 12-Month-Old Guide Dogs
Source: Front Vet Sci. 2016 Jun 22;3:49. doi: 10.3389/fvets.2016.00049 (PMC4916180; doi:10.3389/fvets.2016.00049)
Supplement: Supplementary file 1 [file Table_1.PDF]

Supp. Table 1: Results of Chi-square tests of the relationships between enrvironmental variables (categorical scale) and training outcomes (R=released from training, S=successfully completed training).

**Independent variable**	**N (R/S)**	**Pearson Chi-square**	**df**	**Asymp. Sig. (2-tailed)**
Men in the household	317 / 424	2.389	2	0.303
None	15 / 29			
1 or 2	282 / 361			
3 or more	20 / 34			
Women in the household	320 / 424	4.413	2	0.110
None	0 / 4			
1 or 2	275 / 373			
3 or more	45 / 47			
Children (< 12 years) in the household	321 / 426	0.084	1	0.772
None	206 / 269			
1 or more	115 / 157			
Teenagers (12-18 years) in the household	308 / 404	1.23	1	0.267
None	102 / 150			
1 or more	206 / 254			
Other pets in the home	**321 / 426**	**8.629**	**2**	**0.013**
**None**	**67 / 55**			
**Other dogs**	**195 / 280**			
**Pets other than dogs**	59 / 91			
Fenced-in yard	321 / 426	1.602	1	0.206
Yes	147 / 215			
No	174 / 211			
Puppy ever threatened by an unfamiliar dog	321 / 426	0.628	1	0.428
Yes	85 / 124			
No	236 / 302			
Puppy ever frightened by a familiar or unfamiliar person	**321 / 426**	**4.95**	**1**	**0.026**
**Yes**	**31 / 23**			
**No**	**290 / 403**			

Boldface indicates items used in Generalized Linear Modeling

Supp. Table 2: Results of Mann-Whitney U tests of the relationships between enrvironmental variables (continuous scale) and training outcomes (R=released from training, S=successfully completed training).

	**Test Statistics**				**Released**			**Successful**			**Total**
**Independent variables**	**Mann-Whitney U**	**Wilcoxon W**	**Z**	**Asymp. Sig. (2-tailed)**	**N**	**Mean Rank**	**Sum of Ranks**	**N**	**Mean Rank**	**Sum of Ranks**	**N**
Number of dogs owned in lifetime	**61031**	**111752**	**-2.07**	**0.038**	**318**	**351.42**	**111752**	**421**	**384.03**	**161678**	**739**
Number of previous guide dog puppies raised	**60813**	**112494**	**-2.561**	**0.010**	**321**	**350.45**	**112494**	**424**	**390.07**	**165391**	**745**
Exercise walking outside the house (hours/week)	58598.5	103448.5	-0.569	0.569	299	345.98	103448.5	402	354.73	142602.5	701
Running or playing outside the house (hours/week)	59182.5	105238.5	-0.589	0.556	303	347.32	105238.5	401	356.41	142921.5	704
Time left at home alone (days/week)	61742	109328	-0.293	0.769	308	354.96	109328	406	359.43	145297	714
Time left at home alone (hours/day)	57062	104648	-1.909	0.056	308	339.77	104648	404	369.26	149180	712
Time confined (days/week)	58995	13896	-0.726	0.468	306	358.71	109764	398	347.73	138396	704
Time confined (hours/day)	57270	103326	-0.813	0.416	303	341.01	103326	392	353.4	138534	695

Boldface indicates items used in Generalized Linear Modeling

Supp. Table 3: Results of Mann-Whitney U tests of the relationships between enrvironmental variables (binary) and C-BARQ traits.

	Test Statistics				No			Yes			Total
C-BARQ Traits	Mann-Whitney U	Wilcoxon W	Z	Asymp. Sig. (2-tailed)	N	Mean Rank	Sum of Ranks	N	Mean Rank	Sum of Ranks	N
Other dogs in household											
Stranger-directed aggression	84100	236176	-1.067	0.286	317	444.7	140970	551	428.63	236176	868
**Owner-directed aggression**	66250.5	224453.5	-8.514	**< 0.0001**	319	514.32	164067.5	562	399.38	224453.5	881
**Dog-directed aggression**	71432	203787	-1.99	**0.047**	300	426.39	127918	514	396.47	203787	814
**Dog-directed fear**	69378.5	207979.5	-2.701	**0.007**	294	437.52	128630.5	526	395.4	207979.5	820
Dog rivalry	20019	23179	-1.194	0.232	79	293.41	23179	546	315.84	172446	625
Stranger-directed fear	86284	135739	-0.903	0.366	314	432.29	135739	560	440.42	246636	874
Nonsocial fear	82145	236991	-1.464	0.143	314	451.89	141894	556	426.24	236991	870
Separation-related problems	83207.5	234182.5	-1.086	0.278	317	445.52	141228.5	549	426.56	234182.5	866
**Touch sensitivity**	74836.5	225811.5	-3.253	**0.001**	313	466.91	146141.5	549	411.31	225811.5	862
Fenced-in Yard											
Stranger-directed aggression	93157	188860	-0.323	0.747	437	432.17	188860	431	436.86	188286	868
Owner-directed aggression	92334	188914	-1.639	0.101	442	451.6	199607	439	430.33	188914	881
Dog-directed aggression	80435.5	162245.5	-0.808	0.419	404	401.6	162245.5	410	413.32	169459.5	814
Dog-directed fear	82262	167753	-0.582	0.561	407	414.88	168857	413	406.18	167753	820
Dog rivalry	46414	84089	-0.864	0.387	274	306.89	84089	351	317.77	111536	625
Stranger-directed fear	94649.5	190352.5	-0.442	0.658	437	435.59	190352.5	437	439.41	192022.5	874
Nonsocial fear	88663	183929	-1.625	0.104	434	449.21	194956	436	421.86	183929	870
**Separation-related problems**	85435	177670	-2.28	**0.023**	437	452.5	197741	429	414.15	177670	866
Touch sensitivity	87150.5	179385.5	-1.617	0.106	433	444.73	192567.5	429	418.15	179385.5	862
Teens in the household											
**Stranger-directed aggression**	71428.5	115088.5	-2.208	**0.027**	295	390.13	115088.5	526	422.7	222342.5	821
**Owner-directed aggression**	74156	119006	-2.293	**0.022**	299	398.01	119006	535	428.39	229189	834
**Dog-directed aggression**	61945	102415	-2.839	**0.005**	284	360.62	102415	489	402.32	196736	773
Dog-directed fear	67592	108062	-0.989	0.323	284	380.5	108062	495	395.45	195748	779
Dog rivalry	41083.5	107149.5	-0.273	0.785	229	298.6	68378.5	363	295.18	107149.5	592
Stranger-directed fear	78332.5	122883.5	-0.372	0.710	298	412.36	122883.5	530	415.7	220322.5	828
Nonsocial fear	75038.5	118994.5	-0.959	0.338	296	402.01	118994.5	528	418.38	220905.5	824
Separation-related problems	73313	116384	-1.211	0.226	293	397.22	116384	527	417.89	220226	820
**Touch sensitivity**	65733.5	108804.5	-3.475	**0.001**	293	371.35	108804.5	523	429.31	224531.5	816
Feed puppy before you eat (vs after)											
Stranger-directed aggression	39461.5	45132.5	-0.398	0.690	106	425.78	45132.5	760	434.58	330278.5	866

Supp. Table 3: Results of Mann-Whitney U tests of the relationships between enrvironmental variables (binary) and C-BARQ traits.

	Test Statistics				No			Yes			Total
**C-BARQ Traits**	**Mann-Whitney U**	**Wilcoxon W**	**Z**	**Asymp. Sig. (2-tailed)**	**N**	**Mean Rank**	**Sum of Ranks**	**N**	**Mean Rank**	**Sum of Ranks**	**N**
Owner-directed aggression	41920	338755	-0.024	0.981	109	440.41	48005	770	439.94	338755	879
Dog-directed aggression	34019.5	39375.5	-1.274	0.203	103	382.29	39375.5	709	410.02	290702.5	812
Dog-directed fear	36959	42737	-0.524	0.600	107	399.41	42737	711	411.02	292234	818
Dog rivalry	20202	23205	-0.641	0.521	77	301.36	23205	546	313.5	171171	623
Stranger-directed fear	40597	46592	-0.792	0.428	109	427.45	46592	763	437.79	334036	872
Nonsocial fear	41284.5	47279.5	-0.033	0.973	109	433.76	47279.5	759	434.61	329866.5	868
Separation-related problems	40166	327069	-0.14	0.889	107	435.62	46611	757	432.06	327069	864
Touch sensitivity	38225.5	44111.5	-1.018	0.309	108	408.44	44111.5	752	433.67	326118.5	860
Puppy ever frightened by a person											
**Stranger-directed aggression**	20419.5	343225.5	-2.993	**0.003**	803	427.43	343225.5	63	510.88	32185.5	866
Owner-directed aggression	25767	358287	-0.211	0.833	815	439.62	358287	64	444.89	28473	879
**Dog-directed aggression**	18287	301415	-2.777	**0.005**	752	400.82	301415	60	477.72	28663	812
**Dog-directed fear**	19231	307651	-1.998	**0.046**	759	405.34	307651	59	463.05	27320	818
Dog rivalry	10451	180104	-1.538	0.124	582	309.46	180104	41	348.1	14272	623
**Stranger-directed fear**	20430	347266	-5.526	**< 0.0001**	808	429.78	347266	64	521.28	33362	872
**Nonsocial fear**	21274.5	345689.5	-2.157	**0.031**	805	429.43	345698.5	63	499.31	31456.5	868
Separation-related problems	23364.5	344565.5	-0.99	0.322	801	430.17	344565.5	63	462.13	29114.5	864
**Touch sensitivity**	20011.5	339611.5	-2.404	**0.016**	799	425.05	339611.5	61	501.94	30618.5	860
Puppy ever threatened by an unfamiliar dog											
**Stranger-directed aggression**	66794.5	262419.5	-3.031	**0.002**	625	419.87	262419.5	241	468.84	112991.5	866
**Owner-directed aggression**	72079	273374	-2.185	**0.029**	634	431.19	273374	245	462.8	113386	879
**Dog-directed aggression**	63020.5	227471.5	-2.034	**0.042**	573	396.98	227471.5	239	429.32	102606.5	812
**Dog-directed fear**	59992.5	229063.5	-3.194	**0.001**	581	394.26	229063.5	237	446.87	105907.5	818
Dog rivalry	36243	48646	-0.201	0.841	466	312.73	145730	157	309.85	48646	623
Stranger-directed fear	74821	273586	-0.836	0.403	630	434.26	273586	242	442.32	107042	872
Nonsocial fear	72861	269112	-0.882	0.378	626	429.89	269112	242	446.42	108034	868
**Separation-related problems**	68107.5	261860.5	-2.196	**0.028**	622	421	261860.5	242	462.06	111819.5	864
**Touch sensitivity**	67468	260599	-2.131	**0.033**	621	419.64	260599	239	458.71	109631	860

Boldface indicates variables used in Generalized Linear Modeling.

Supp. Table 4: Results of Kruskal Wallis tests of the relationships between enrvironmental variables (categorical scale) and C-BARQ traits.

	Test Statistics				None		Other dogs		Pets other than dogs		Total
Dogs and other pets in the home	**Chi-Square**	**df**	**Asymp. Sig.**		**N**	**Mean Rank**	**N**	**Mean Rank**	**N**	**Mean Rank**	**N**
Stranger-directed aggression	1.936	2	0.38		148	432.52	549	427.57	169	453.63	866
**Owner-directed aggression**	72.219	2	**<0.0001**		148	507.46	560	398.47	171	517.63	879
Dog-directed aggression	4.632	2	0.099		140	415.52	512	395.38	160	434.19	812
**Dog-directed fear**	8.098	2	**0.017**		136	449.68	524	394.62	158	424.25	818
Dog rivalry	1.609	2	0.447		28	303.32	544	314.81	51	286.75	623
Stranger-directed fear	2.096	2	0.351		147	439.78	558	439.48	167	423.66	872
Nonsocial fear	2.14	2	0.343		146	447.36	554	425.35	168	453.51	868
Separation-related problems	1.979	2	0.372		147	457.29	547	425.16	170	434.68	864
**Touch sensitivity**	11.595	2	**0.003**		147	482.05	547	410.62	166	450.36	860

	Test Statistics				None		1 or 2		3 or more		Total
Women in the household	**Chi-Square**	**df**	**Asymp. Sig.**		**N**	**Mean Rank**	**N**	**Mean Rank**	**N**	**Mean Rank**	**N**
Stranger-directed aggression	2.796	2	0.247		5	279	749	433.16	107	422.95	861
Owner-directed aggression	0.274	2	0.872		5	431.3	759	436.26	110	446.35	874
Dog-directed aggression	1.169	2	0.557		5	318.5	705	403.07	97	415.18	807
Dog-directed fear	1.992	2	0.369		5	431.4	710	402.99	98	434.79	813
Dog rivalry	2.619	2	0.270		2	196	525	313.58	92	292.07	619
Stranger-directed fear	0.525	2	0.769		5	393	753	434.31	109	433.75	867
Nonsocial fear	1.095	2	0.578		5	320.2	751	431.88	107	438.05	863
Separation-related problems	5.677	2	0.059		5	419.4	747	422.96	108	483.18	860
Touch sensitivity	1.00	2	0.607		5	377.8	743	425.55	107	447.39	855

	Test Statistics				None		1 or 2		3 or more		Total
Men in the household	**Chi-Square**	**df**	**Asymp. Sig.**		**N**	**Mean Rank**	**N**	**Mean Rank**	**N**	**Mean Rank**	**N**
Stranger-directed aggression	3.642	2	0.162		62	424.53	735	426.3	62	479.35	859
Owner-directed aggression	1.782	2	0.410		63	432.73	746	434.22	63	467.25	872
Dog-directed aggression	2.088	2	0.352		60	396.05	686	400.44	59	439.83	805
Dog-directed fear	0.186	2	0.911		62	395.94	692	406.42	57	411.82	811
Dog rivalry	5.403	2	0.067		56	331.32	518	303.93	45	353.29	619
Stranger-directed fear	5.642	2	0.060		62	426.6	740	430.45	63	469.21	865
Nonsocial fear	0.915	2	0.633		62	413.63	738	430.44	61	455.39	861
Separation-related problems	2.065	2	0.356		63	467.7	732	427.46	62	407.85	857
Touch sensitivity	5.28	2	0.071		61	359.34	730	432.34	62	430.65	853

Boldface indicates variables used in Generalized Linear Modeling.

Supp. Table 5: Results of Spearman's correlation tests of the relationships between enrvironmental variables (continuous scale) and C-BARQ traits.

	**Test Statistics**		
Number of dogs owned during lifetime	**Spearman's rho**	**Sig. (2-tailed)**	**N**
Stranger-directed aggression	-0.052	0.130	857
**Owner-directed aggression**	-0.145	**<0.0001**	870
Dog-directed aggression	-0.068	0.054	803
Dog-directed fear	-0.058	0.098	809
Dog rivalry	0.061	0.128	615
Stranger-directed fear	-0.019	0.582	863
Nonsocial fear	-0.036	0.296	860
**Separation-related problems**	-0.08	**0.019**	855
**Touch sensitivity**	-0.131	**<0.0001**	851

Number of guide dog puppies raised	**Spearman's rho**	**Sig. (2-tailed)**	**N**
**Stranger-directed aggression**	-0.113	**0.001**	864
**Owner-directed aggression**	-0.245	**<0.0001**	877
**Dog-directed aggression**	-0.151	**<0.0001**	810
**Dog-directed fear**	-0.129	**<0.0001**	816
Dog rivalry	-0.076	0.058	622
Stranger-directed fear	-0.025	0.470	870
**Nonsocial fear**	-0.114	**0.001**	866
**Separation-related problems**	-0.149	**<0.0001**	862
**Touch sensitivity**	-0.236	**<0.0001**	858

Boldface indicates items used in Generalized Linear Modeling
