# Supplementary material for: Aspects of Juvenile and Adolescent Environment Predict Aggression and Fear in 12-Month-Old Guide Dogs
Source: Front Vet Sci. 2016 Jun 22;3:49. doi: 10.3389/fvets.2016.00049 (PMC4916180; doi:10.3389/fvets.2016.00049)
Supplement: Supplementary file 2 [file Data_Sheet_1.DOCX]

PUPPY RAISER FINAL REPORT

Puppy’s Name: ____________________ Breed: _______________ Tattoo#: __________

Your Name: __________________________________Age______ Today’s Date: __________

1. Area of residence: country city suburbs

2. Housing situation. Please check one.

⬜ Apartment ⬜ Condominium ⬜ Row house ⬜ Duplex

⬜ Townhouse ⬜ House ⬜ Farm

3. Which of the following best describes the road(s) or street(s) closest to your home?

⬜ Quiet country roads (very low traffic volume)

⬜ Country roads or suburban side streets (low traffic volume)

⬜ Smaller highways or town/city streets (moderate traffic volume)

⬜ Busy highways, main streets, or expressways (heavy traffic volume)

4. Does your puppy have access to a fenced-in yard?

⬜ YES ----- How often do you let the SE puppy out in the yard? _____ hours/day

⬜ NO

5. Are there stairs/staircases in your home that your puppy used on a regular basis?

⬜ YES ⬜ NO

6. How many people in the household?

No. of adults in household:_______ (____men _____women)

No. of children in household: _______ Ages:________________________

7. Do you currently own a dog(s)? ⬜ YES ⬜ NO

7a. If YES, please answer the following questions. If NO, please skip to 7b

7a. 1. How old is (are) the other dog(s)? __________, Sex:________, Breed:_______________

__________, Sex:________, Breed:_______________

__________, Sex:________, Breed:_______________

7a. 2. Would you be willing to fill out a behavioral questionnaire for your other dog(s)?

⬜ YES ⬜ NO

7b. If you don’t currently own other dogs, when did you last own a dog (including previous guide dog puppy, if appropriate)? Please check one.

______Less than 6 months ago

______Within the last year

______Within the last 5 years

______More than 5 years ago

______Never

8. Roughly how many dogs do you recall owning/living with in your whole life (excluding Seeing Eye Puppies)? ____________?

9. How many guide dog puppies (if any) have you/your family raised previously? _________

10. How old were you when you/your family first owned a dog?_______years old______

11. Do you currently own cats?

⬜ YES----How many cats do you recall owning/living with in your whole life?______

⬜ NO-----Have you ever owned cats?

⬜ YES – when did you last own a cat________

⬜ NO

12. Do you currently own any pets other than cats or dogs?

⬜ YES

⬜ NO -----Have you ever owned pets other than cats or dogs?

⬜ YES – when did you last own pets other than cats or dogs _____

⬜ NO

13. Did you move while raising your puppy?

⬜ YES If yes how old was your puppy______

⬜ NO

13a. Has your puppy always lived with you?

⬜ YES

⬜ NO If no age of puppy when you received it_______

14. Where does your puppy usually sleep at night?

⬜ In a bedroom, in its own bed or crate.

⬜ Tied down beside someone’s bed.

⬜ Elsewhere in house, but not confined

⬜ Elsewhere in house, but confined to room or crate.

⬜ Outside in yard or kennel

⬜ In someone’s bedroom, on bed

# 15. Eating habits

Type of food_____________________________________________________

Number of feedings _________amount of food________________the kibble ⬜ dry ⬜ moist

My puppy was always a ⬜ good eater ⬜ sometimes picky ⬜ always picky

My puppy ate quickly (within 5 minutes) ⬜ yes ⬜ no

When do you usually feed your SE puppy? ⬜ Before or ⬜ After you eat

16. Housebreaking

My puppy was easy to housebreak ⬜ yes ⬜ no

S/he ⬜ did ⬜ did not have any accidents in the last month ⬜ Urinary ⬜ Bowel

My puppy took care of his/her needs while on a leash at home ⬜ yes ⬜ no, on a walk ⬜ yes ⬜ no,

Loose in fenced yard ⬜ yes ⬜ no

Some pups urinate a little if excited or afraid. In the past few months, mine ⬜ did ⬜ did not

Comments:

17. How much exercise does your puppy usually get?

Walking outside of the house: __________hours/week

Running/playing outside of the house: __________hours/week

18. Please indicate how many days/week and how much time/day your puppy is usually left at

home alone. _____ days/week, _____ hours/day

19. Please indicate roughly how many days/week and how much time/day your puppy is usually

confined. _____ days/week, _____ hours/day.

The method of confinement:

⬜ in a crate

⬜ on a leash or chain

⬜ confined in a specific room/space: which room? __________

20. If you currently own any dog(s) other than this puppy, please answer the following questions. If not, please skip to Q. 21?

20a. Which of the following best describes the relationship between the puppy and your other dog(s)?

⬜ Affectionate: always friendly and/or playful together.

⬜ Competitive: usually friendly and/or playful, but they may growl/bark at each other in competitive situations.

⬜ Neutral/indifferent; they tend to ignore each other most of the time.

⬜ One-sided; the SE puppy is friendly/playful, but my other dog(s) is aloof, aggressive, or fearful (please specify which by underlining).

⬜ One-sided; my other dog(s) is friendly/playful, but the SE puppy is aloof, aggressive, or fearful (please specify which by underlining).

⬜ Hostile; the dogs tend to avoid and/or threaten each other.

21. Has your guide dog puppy ever been threatened (chased, growled or barked at, bitten, etc.) by an unfamiliar dog?

⬜ NO

⬜ YES ----- Roughly, how old was your SE puppy when it was threatened by an unfamiliar dog for the first time?

⬜ < 3 months old ⬜ 3-6 months old ⬜ 6-9 months old ⬜ > 9 months old

Please describe the incident(s): ______________________________________

Comments:

22. Has your puppy ever been frightened by a familiar or unfamiliar person?

⬜ NO

⬜ YES ----- Roughly, how old was your SE puppy when it was frightened by a person for the first time?

⬜ < 3 months old ⬜ 3-6 months old ⬜ 6-9 months old ⬜ > 9 months old

Please describe the incident(s): ______________________________________

Comments:

23. Barking and/or growling

(Check all that apply) Barks Growls Explanation (if sometimes, why)

If visitors come to my home ⬜ ⬜ ___________________________________

When meeting strangers ⬜ ⬜ ___________________________________

When s/he sees another dog ⬜ ⬜ ___________________________________

When s/he sees an animal ⬜ ⬜ ___________________________________

When s/he is in the car ⬜ ⬜ ___________________________________

When s/he is in the yard ⬜ ⬜ ___________________________________

When s/he sees children ⬜ ⬜ ___________________________________

When s/he is left alone ⬜ ⬜ ___________________________________

When doorbell rings ⬜ ⬜ ___________________________________

24. During the last few months how often has your puppy encountered the following events or experiences (e.g. Infrequently = once or twice; Occasionally = approximately monthly; Frequently = approximately weekly; Very Frequently = several times per week or more).

In column B, please also score the puppy’s typical reaction to each event/experience (1=never observed; 2=fearful; 3=startles, recovers; 4=secure, confident; 5=ignores).

B

|  |  |  |  |  | Very |  |
| --- | --- | --- | --- | --- | --- | --- |
|  | Never | Infrequently | Occasionally | Frequently | Frequently | REACTION |
| Unfamiliar adults | ⬜ | ⬜ | ⬜ | ⬜ | ⬜ |  |
| Unfamiliar children | ⬜ | ⬜ | ⬜ | ⬜ | ⬜ |  |
| Unfamiliar dogs | ⬜ | ⬜ | ⬜ | ⬜ | ⬜ |  |
| Unfamiliar cats | ⬜ | ⬜ | ⬜ | ⬜ | ⬜ |  |
| Familiar dogs | ⬜ | ⬜ | ⬜ | ⬜ | ⬜ |  |
| Familiar cats | ⬜ | ⬜ | ⬜ | ⬜ | ⬜ |  |
| Birds | ⬜ | ⬜ | ⬜ | ⬜ | ⬜ |  |
| Squirrels | ⬜ | ⬜ | ⬜ | ⬜ | ⬜ |  |
| Farm animals/ livestock | ⬜ | ⬜ | ⬜ | ⬜ | ⬜ |  |
| Lawn mowers | ⬜ | ⬜ | ⬜ | ⬜ | ⬜ |  |
| Thunder | ⬜ | ⬜ | ⬜ | ⬜ | ⬜ |  |
| Sudden loud noises  (bangs, crashes, etc.) | ⬜ | ⬜ | ⬜ | ⬜ | ⬜ |  |
| Traffic sounds | ⬜ | ⬜ | ⬜ | ⬜ | ⬜ |  |
| Busy traffic | ⬜ | ⬜ | ⬜ | ⬜ | ⬜ |  |
| Heavy trucks | ⬜ | ⬜ | ⬜ | ⬜ | ⬜ |  |
| Cars honking | ⬜ | ⬜ | ⬜ | ⬜ | ⬜ |  |
| Trains | ⬜ | ⬜ | ⬜ | ⬜ | ⬜ |  |
| Crowds | ⬜ | ⬜ | ⬜ | ⬜ | ⬜ |  |
| Slippery floors | ⬜ | ⬜ | ⬜ | ⬜ | ⬜ |  |
| Stairs/staircases | ⬜ | ⬜ | ⬜ | ⬜ | ⬜ |  |
| Sidewalk grates | ⬜ | ⬜ | ⬜ | ⬜ | ⬜ |  |
| Car rides | ⬜ | ⬜ | ⬜ | ⬜ | ⬜ |  |
| Elevators | ⬜ | ⬜ | ⬜ | ⬜ | ⬜ |  |

Comments:
